# Drug resistance in metastatic castration-resistant prostate cancer: an update on the status quo

**DOI:** 10.20517/cdr.2022.15

**Published:** 2022-06-22

**Authors:** Amani Yehya, Fatima Ghamlouche, Amin Zahwe, Yousef Zeid, Kevork Wakimian, Deborah Mukherji, Wassim Abou-Kheir

**Affiliations:** ^1^Department of Anatomy, Cell Biology and Physiological Sciences, Faculty of Medicine, American University of Beirut, Beirut 1107-2020, Lebanon.; ^2^Division of Hematology/Oncology, Faculty of Medicine, American University of Beirut Medical Center, Beirut 1107-2020, Lebanon.; ^#^Equally contributing authors.

**Keywords:** Prostate cancer, mCRPC, androgen receptor, drug resistance, novel targeted therapeutics

## Abstract

Prostate cancer (PCa) is a leading cause of cancer-related morbidity and mortality in men globally. Despite improvements in the diagnosis and treatment of PCa, a significant proportion of patients with high-risk localized disease and all patients with advanced disease at diagnosis will experience progression to metastatic castration-resistant prostate cancer (mCRPC). Multiple drugs are now approved as the standard of care treatments for patients with mCRPC that have been shown to prolong survival. Although the majority of patients will respond initially, primary and secondary resistance to these therapies make mCRPC an incurable disease. Several molecular mechanisms underlie the development of mCRPC, with the androgen receptor (AR) axis being the main driver as well as the key drug target. Understanding resistance mechanisms is crucial for discovering novel therapeutic strategies to delay or reverse the progression of the disease. In this review, we address the diverse mechanisms of drug resistance in mCRPC. In addition, we shed light on emerging targeted therapies currently being tested in clinical trials with promising potential to overcome mCRPC-drug resistance.

## INTRODUCTION

Prostate cancer (PCa) is a leading cause of mortality and morbidity among men worldwide, where 12.5% of men are prone to be diagnosed with PCa during their lifetime^[1]^. According to the Globocan data collected from 185 countries in 2020, an estimated 1.4 million new PCa cases were diagnosed, with an incidence rate of 37.5 per 100,000 males, accounting for 14.1% of all male cancer diagnoses. In addition, 375,000 deaths due to PCa were recorded, comprising 6.8% of cancer deaths in men^[2]^. PCa was discovered to be androgen-sensitive by Huggins and Hodges in 1941^[3]^. The activation of androgen receptor (AR)-mediated signaling is an essential route for regulating PCa cell growth and proliferation; thereby, alterations in this cascade hold a great influence on cancer progression^[4]^. This relation between androgen and PCa leads to androgen deprivation treatment (ADT), which includes surgical castration through orchiectomy to remove the source of androgen or medical castration through antiandrogen and gonadotropin-releasing hormone analogs, thus reducing androgens to castrate levels^[3]^. Interventions such as prostatectomy or radiotherapy along with ADT for localized PCa are the standard of care for localized disease, yet ADT remains the backbone of first-line treatment for locally advanced and metastatic PCa^[5]^. For patients treated with long-term gonadal androgen suppression, PCa cells become resistant to ADT within 2-3 years and the malignancy progresses even when the serum testosterone level is below the castrate level. This stage of prostate cancer, known as castration-resistant prostate cancer (CRPC), can progress to the lethal form of metastatic CRPC (mCRPC), resulting in rapid mortality with mean overall survival historically of 16-18 months^[6]^.

Although curative treatment has not yet been reached, multiple therapeutic modalities for mCRPC patients have been accomplished^[7-16]^ [Table 1].

**Table 1:** Summary table of the drugs and agents indicated for mCRPC

**Class**	**Agent**	**MOA**	**Studies enrolled with indications**	**Trial result**
**Hormonal therapy**	Abiraterone Acetate	Irreversible selective inhibitor of CYP17-alpha-hydroxylase and C17, 20-lyase coupled with a modest AR antagonist activity^[7]^	COU-AA-301; post-docetaxel^[8]^	ABI increased median overall survival when compared to the placebo group by 3.9 months
			COU-AA-302; pre-docetaxel^[9]^	ABI significantly increased median overall survival when compared to the placebo group by 8.2 months
**AR antagonists**	Enzalutamide	AR antagonist impedes nuclear receptor translocation and DNA binding, induces apoptosis	AFFIRM; post-docetaxel^[10]^	Overall survival increased by 4.8 months in comparison to the control group
	Darolutamide		ARCADES^[11]^	86% of patients treated with 1400 mg dose of darolutamide had a 50% or greater decrease in PSA
**Cytotoxic chemotherapy**	Docetaxel	Inhibits microtubular depolymerization arresting their function	TAX 327; first-line chemotherapy^[12]^	DOC combined with prednisone increased overall survival by 2.9 months when compared to the control group treated with mitoxantrone plus PDN
	Cabazitaxel		TROPIC, Phase III, randomized, open-label^[13]^	Overall survival increased by 2.4 months in the treatment group (CBZ+ PDN) compared to patients treated with a combination of PDN and mitoxantrone
**Calcium-mimetic**	Ra-223	Emits high energy alpha particles after adhering selectively to regions of elevated bone turnover	ALSYMPCA, Phase III, randomized^[14]^	Patients placed on Ra-223 treatment had 3.6 months increase in survival compared to the placebo group
**PARP inhibitor**	Olaparib	Impede PARP causing cumulative DNA and cell damage	PROfound Phase III, randomized^[15]^	Patients with mutations in *BRCA1*, *BRCA2*, or *ATM *genes assigned to receive olaparib experienced a 4.4-month increase in survival compared to the control arm receiving ENZ or ABI plus prednisone
**Dendritic-cell vaccine**	Sipuleucel-T	Infused within APCs to present PAP peptides *in vivo* activating CD4+ and CD8+ T cells	IMPACT trial, Phase III^[16]^	Utilizing sipuleucel-T extended overall survival among men with mCRPC by 10 months

MOA: Mode of action; AR: androgen receptor; CYP17: Cytochrome P450 enzyme 17; PSA: prostate-specific antigen; ABI: abiraterone; DOC: docetaxel; PDN: prednisone; CBZ: cabazitaxel; ENZ: enzalutamide; PARP: poly(ADP-ribose) polymerase; APC: antigen-presenting cells; PAP: prostatic acid phosphatase.

### Inhibitors of CYP17

The emerging speculative conveyance regarding the defiance of castration resistance classes to androgen manipulating therapies has been fully debunked recently, thus reemphasizing the chief role of the androgen signaling axis as a subject to therapy development^[17]^. Cytochrome P450 enzyme 17 (CYP17) has been recognized as a vital mediator in steroidal biosynthesis, especially androgen. To impede this vicious pathway, several drugs, such as antifungal ketoconazole, were depicted as potential options; however, prohibitive toxicities paired with minute survival benefits raised the need for better alternatives^[18]^. Abiraterone (ABI) acetate functions as an irreversible selective steroidal agent for the microsomal complex CYP17, thus restraining two functionalities: cortisol biosynthesis in the adrenal gland (via C21 17-α-hydroxylation) and steroid production in testis and adrenal gland (lyase property)^[19]^. As a result, both testosterone supply and cortisol will be inhibited, causing a flux of augmented steroid levels upstream of the target halt. Therefore, prednisone (PDN), a corticosteroid that binds to the glucocorticoid receptor (GR), is often co-administered with ABI, seeking in return a diminished side effect for mineralocorticoid excess syndrome^[20]^. Significantly ameliorated radiographic progression-free survival (rPFS) and overall survival were reported in a large Phase III trial for asymptomatic CRPC patients treated with ABI (Abiraterone) and PDN compared to PDN alone (16.5 *vs.* 8.2 months; HR: 0.52; 95%CI: 0.45-0.61; *P* < 0.0001)^[9]^. Based on the data presented by such trials, the FDA approved ABI as a management therapy for CRPC patients. Subsequently, the drug has also been shown to improve survival in patients with hormone-sensitive metastatic disease and is also approved as first-line therapy in combination with ADT^[21]^.

### AR antagonists 

The oral AR antagonist enzalutamide (ENZ) lashes with high affinity, impeding nuclear receptor translocation and DNA binding, thereby inducing apoptosis^[22]^. Based on the results of two Phase III placebo-controlled studies where a 4.8-month median overall survival benefit was reported in chemotherapy-naïve mCRPC patients, both the FDA and EMA ratified ENZ usage for mCRPC previously treated with docetaxel^[23]^. Notably, the steroidal biosynthesis impact was nullified with ENZ utilization, thus shifting away from PDN prescription during the treatment regimen^[24]^.

### Cytotoxic chemotherapy 

By reversibly stabilizing the microtubules and promoting their assembly, docetaxel (DOC) fosters a mitotic block on cancerous cells. After augmentation of these bundles, the natural dynamics of mitosis would be impaired, consequently leading to apoptosis, which is further induced by phosphorylation of the Bcl-2 oncoprotein^[25]^. Furthermore, impaired microtubules functionality surges p53 buildup in the nucleus, thus driving its downstream signaling forward^[26]^. Notably, due to their impact on microtubules, it has been shown that taxanes also possess antiangiogenic capabilities paired with impeding AR trafficking and accumulation^[27]^. This drug was granted FDA and EMA approval based on the results of the TAX 327 study conducted on patients diagnosed with CRPC and subjected to DOC treatment paired with PDN compared to the standard approach of mitoxantrone and PDN in the control arm. The results demonstrate an overall increased survival benefit (19.2 months *vs.* 16.3 months; *P* < 0.004) reinforced with significant amelioration in pain relief^[27]^. The incorporation of PDN augments the efficacy of DOC and results in superior outcomes as a result of the additive benefit from the two drugs, each known to be effective in PCa through targeting different signaling, AR and GR. In addition, prednisone also has anti-inflammatory properties and can treat pain, nausea, and edema^[28-30]^. Moreover, the cytotoxic tubulin-binding drug cabazitaxel (CBZ) has demonstrated a prevailing advantage in enhancing the survival of patients with mCRPC after being assigned to a DOC-based treating regimen, causing a 30% decline in risk of death when compared to those treated with mitoxantrone (panel). These results granted CBZ US Food and Drug Administration approval for being prescribed as a second-line treatment in mCRPC^[13]^.

## DRUG RESISTANCE IN mCRPC

**Figure 1:**
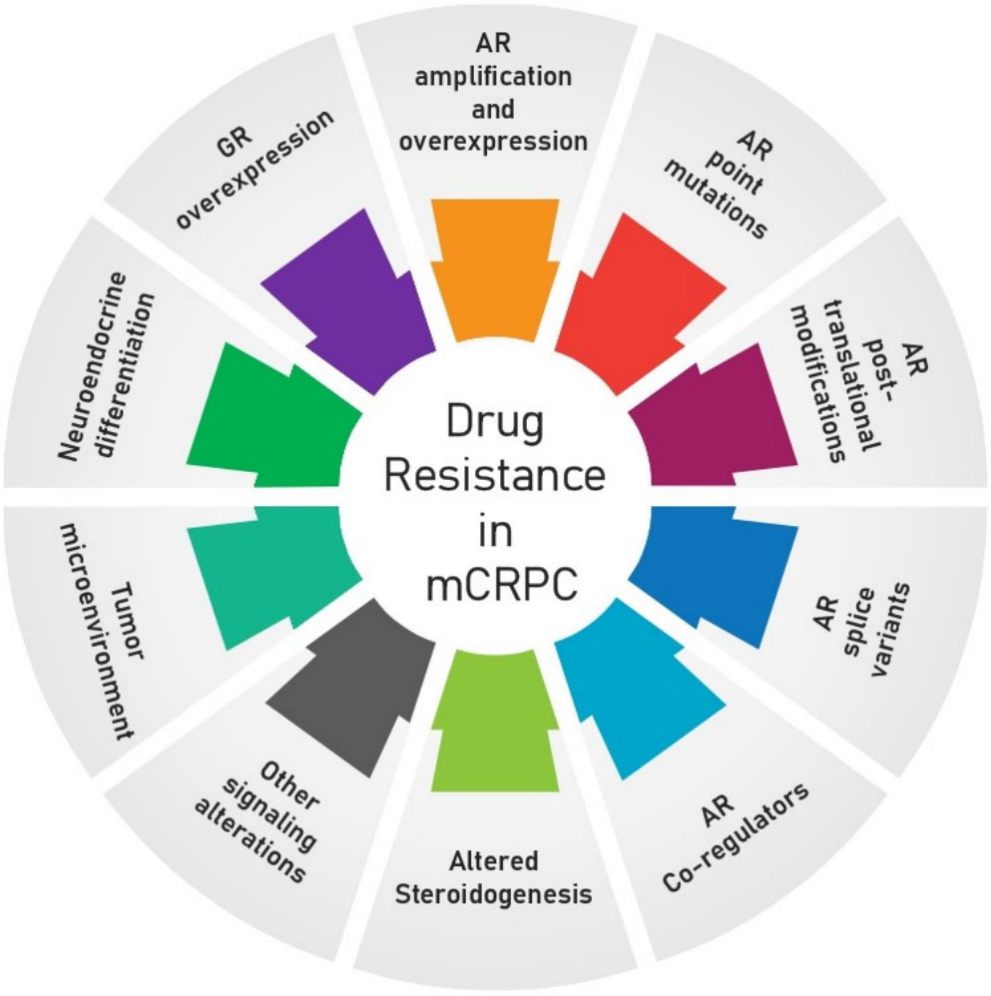
Mechanisms of drug resistance in mCRPC. Several mechanisms of drug resistance are well defined in CRPC, including AR amplification and overexpression, AR point mutations, AR post-translational modifications, AR splice variants, AR co-regulators, altered steroidogenesis, GR overexpression, neuroendocrine differentiation, tumor microenvironment, and other signaling alterations. AR: Androgen receptor; GR: glucocorticoid receptor; mCRPC: metastatic castration-resistance prostate cancer.

Despite the significant survival benefit of the currently approved therapies, which can alleviate symptoms and prolong overall survival, mCRPC remains incurable as primary and secondary resistance develops rapidly^[31]^. Drug resistance can develop due to mechanisms intrinsic to the biology of PCa or by more general mechanisms shared with diverse tumor types, as can be seen in Figure 1*.*

### AR amplification and overexpression

AR gene amplification has been recognized in clinical studies as the most common genetic alteration deriving AR reactivation and progression to CRPC^[32]^. AR overexpression can result from different mechanisms such as gene amplification, increased histone acetylation/phosphorylation at enhancers sites, overexpression of co-regulators, or enhanced protein stability^[33,34]^. The increase in AR expression was found to be consistently linked with the development of resistance to ADT, resulting in PCa progression from a castration-sensitive phenotype to a castration-resistant one. This increase is capable of sensitizing PCa cells to castrate concentrations of androgen and converting the action of AR antagonists to agonists^[24,35]^.

Among patients with CRPC, up to 80% exhibit a significant upregulation in AR transcripts and protein levels^[36]^, in comparison to < 1% of the primary androgen-dependent PCa cases^[37]^. This aberration has been detected, at high frequency, in circulating tumor cells (CTCs) and circulating tumor DNA (ctDNA) and is more common in pretreated CRPC patients than in treatment-naïve patients. A study using liquid biopsies and ctDNA showed that 50% of CRPC patients, pretreated with either ENZ or orteronel (a CYP17A1 inhibitor), evidenced AR amplification^[38,39]^. One *in vivo* study utilizing CRPC xenografts treated with ABI showed a three-fold increase in AR expression^[40]^. In a separate *in vitro* study, a bicalutamide-resistant LNCaP cell line was shown to display an overexpressed AR and hyper-sensitivity to minimal levels of androgen^[41]^. *In vitro* studies using ENZ-resistant LNCaP cells showed an increase in AR levels compared to naïve LNCaP cells^[42]^.

### AR point mutations

AR point mutations are also more frequent in CRPC than in primary androgen-sensitive PCa, especially among patients pretreated with ADT. Several studies evidenced somatic AR point mutations in ~10% of CRPC tissues, whereas none were found in any of the primary PCa tissues examined^[36,37,43]^. This is consistent with results obtained in a previous study that performed a targeted AR sequencing in a cohort of 181 primary cancers and 37 CRPCs. The study revealed that somatic AR point mutations were found to occur only in CRPC and more frequently in patients subjected to prior antiandrogen treatments^[44]^. Supposedly, when AR signaling is more effectively suppressed, clonal selection of tumor cells will enhance AR somatic mutations, subsequently yielding aberrant transcriptomes^[43]^. There are > 100 clinically-relevant somatic point mutations detected in AR^[44]^; the majority are single-base substitutions and are clustered in the ligand-binding domain (LBD). The modifications in LBD alter the steroid-binding pocket and result in broadening ligand specificity and AR activation by alternative non-androgen ligands including progesterone, estrogen, and some AR antagonists^[45]^.

Four main recurrent LBD point mutations are described in several studies: (1) L702H, a leucine to histidine substitution at amino acid 702; (2) W742C, a tryptophan to cysteine substitution at amino acid 742; (3) H875Y, a histidine to tyrosine substitution at amino acid 875; and (4) T878A, a threonine to alanine substitution at amino acid 878 (T878A). These mutations are present in approximately 15%-20% of CRPC cases^[46,47]^. The gain of function mutation “T878A” described in the LNCaP cell line was shown to confer an expansion of binding specificity to AR by both steroid hormones (e.g., progesterone) and first-generation antiandrogens (e.g., bicalutamide or flutamide)^[48,49]^. L702H, a mutation that causes glucocorticoid-mediated AR activation, is associated with primary resistance to ABI^[50]^. In another study, patients harboring T878A and L702H mutations showed poor prostate-specific antigen (PSA) response after ABI or ENZ treatment^[38]^. In addition, several AR mutations were shown to be capable of converting the AR antagonists into agonists, as seen with F877L and F876L mutations associated with resistance to second-generation antiandrogens ENZ and ARN-509^[50,51]^. In line with other studies, the data obtained validate the significance of AR mutations in deriving drug resistance.

### AR post-translational modifications

AR post-translational modifications (PTMs), including serine/threonine and tyrosine phosphorylation, acetylation, methylation, ubiquitination, and sumoylation, have essential roles in enhancing AR activity through maintaining protein stability, nuclear localization, and transcriptional activity^[52,53]^. Thereby, PTMs may contribute to AR reactivation and acquisition of drug resistance in mCRPC^[54]^. A study indicated that the usage of hypomethylating drugs has the effect of reversing and delaying progression to mCRPC. One potential underlying mechanism is through downregulating DNA methyltransferase 1-dependent STAT3 activity^[55]^. Phosphorylation of AR residues plays a significant role in activating AR and promoting continued PCa cell growth. The treatment of the castrate-recurrent CWR-R1 cell line with EGF under low androgen conditions was shown to promote AR transcriptional activity through the phosphorylation of AR at serine 515 and serine 578, MAPK, and PKC consensus sites, respectively^[56]^. A prostate tissue microarray analysis indicated the intracellular non-receptor tyrosine kinase (NRTK) Ack1 to induce AR phosphorylation at Tyrosine 284 and correlate positively with disease progression and negatively with the survival of PCa patients. In activated Ack1-expressing prostate cells, treatment with antiandrogens failed to affect the expression and activation of AR^[57]^. In addition, the NTRK SRC proved clinically to be at high levels in mCRPC and to mediate AR tyrosine phosphorylation and subsequent pathway activation^[58-61]^.

Ubiquitination is another PTM that was shown to have an important role in regulating AR activity. RNF6, a ubiquitin ligase with an AR enhancing role, acts by inducing AR ubiquitination and promoting AR transcriptional activity. RNF6 was detected t at elevated levels in hormone-refractory human PCa tissues^[62]^. Another study identified ubiquitin-specific protease 12 (Usp12) as a positive regulator of AR. Usp12, in complex with the cofactors Uaf-1 and WDR20, interacts with and deubiquitinates AR, resulting in increased protein stability and transcriptional activity of AR^[63]^.

### AR co-regulators: co-activators, co-repressors, and chromatin remodelers

A series of co-regulator protein factors such as co-activators, co-repressors, and chromatin remodelers are known to regulate AR transcriptional activity by being co-recruited to chromatin and binding directly or in a protein complex to AR to regulate gene activity. The balance of co-activator and co-repressors is required for the proper regulation of AR-mediated transcription. Misbalance in these factors leads to higher AR activity and less active antagonism by antiandrogens contributing to drug resistance and mCRPC progression^[64,65]^. FKBP51, a co-activator and a target gene of AR, was detected at upregulated levels in relapsed LAPC-4 tumors grown in castrated mice^[66]^. FKBP51 improved the formation of a super chaperone complex via recruiting p23 co-chaperone to ATP-bound Hsp90, which in line keeps AR with a high-affinity conformation for ligand-binding, consequently promoting androgen-mediated AR transcriptional activity and growth^[66,67]^.

Elevation of AR signaling can also occur through the loss of AR regulatory repressing signals. For example, retinoblastoma (Rb), a well-known tumor suppressor, was shown to be highly reduced in mCRPC and to be associated with tumor recurrence. The loss or inactivation of Rb induced an E2F1-mediated increase in the levels of AR mRNA/protein and two relevant target genes, *PSA* and *TMPRSS2*, consequently promoting castrate-resistant growth and resistance to bicalutamide in PCa cells^[68]^.

A recent study identified and verified myosin heavy chain 9 (MYH9) as a novel AR cofactor. The data show that inhibiting MYH9 in an androgen-independent cell line (LNCaP-AI) promoted AR nuclear translocation and enhanced the expression of PSA, indicative of the MYH9 role as a co-repressor to nuclear AR signaling and a novel factor mediating the progression of CRPC^[69]^.

Other pioneering factors, i.e., FOXA1 and GATA2, act as chromatin remodelers at enhancer sites to regulate the expression and activity of AR in mCRPC. FOXA1 induces open chromatin conformation to allow the binding of other transcription factors, aiding in an enhanced AR transactivation and mCRPC progression^[70]^. CTCs isolated from patients with ABI- or ENZ-resistant metastasis showed amplification in the FOXA1 gene in > 30% of CRPC patients, pinpointing the crucial role of FOXA1 in AR regulation and tumor progression^[71]^. In addition, GATA2 was shown to boost AR activity and CRPC progression. Overexpression of GATA2 was correlated with more aggressiveness in PCa^[72]^. High expression of GATA2 in CRPC cell line “ARCaPM” brings about an increased IGF-2 expression and consequently confers chemotherapy resistance^[73]^. An additional important AR co-regulator and chromatin remodeling factor is homeobox B13 (HOXB13), which acts by interacting with AR and binding to its target loci^[74]^. Analysis of transcriptome expression from several databases revealed that the expression of HOXB13 is elevated during the progression of the primary PCa to CRPC^[75]^. In a separate study, HOXB13 was shown to be highly expressed in hormone-refractory tumors compared to tumors without PSA after initial treatment^[76]^. Interestingly, HOXB13 also mediates the oncogenic function of AR splice variants, mainly ARV7, and acts as a pivotal upstream regulator of AR-V7–driven transcriptomes^[77]^. These findings could explain HOXB13’s role in resistance towards antiandrogen treatment.

### AR splice variants

Transcriptionally activated AR splice variants (ARVs) are truncated forms of AR that lack the C-terminal LBD region, the binding site for first- and second-generation drugs, hence remain constitutively activated independently of androgen or antagonist action. This ligand-independence feature makes ARVs potentially contributors to disease progression and treatment resistance in mCRPC. ARV1, ARV7, and ARV567 are the most common isoforms, while ARV7 is the most extensively studied one^[78,79]^. ARV7 is associated with resistance to both ABI and ENZ. Studying ARV7 expression in CTCs revealed its association with clinical resistance to both drugs in men receiving ENZ. Patients with detectable ARV7 levels had poorer PSA response, shorter progression-free survival (PFS), and reduced overall survival (OS) compared to ARV7-negative patients. Similar results were observed in patients treated with ABI. Markedly, the ARV7 level was higher in males pretreated with ENZ and ABI than in treatment-naïve males^[80]^. The selective siRNA-mediated knockdown of ARV7 in CWR22Rv1 cells inhibited androgen-independent growth and re-established the responsiveness to antiandrogen drugs^[81]^. In one study, ARV567es was identified and characterized as a contributor to PCa progression and resistance to castration in human PCa xenograft models and a biomarker for patients with early recurrence. In addition, AR-V567es transcripts detected in 23% of CRPC bone metastases were shown to be associated with poor prognosis and short survival^[82]^. These data strongly support the implication of ARVs as resistance-driving factors in CRPC.

### Intra-tumoral and alternative androgen biosynthesis/altered steroidogenesis

Although CRPC develops in the presence of sub-physiological levels of circulating androgens, its progression is associated with the accumulation or synthesis of intra-tumoral androgen and accordingly maintained AR activity^[83]^. The low plasma levels of androgens following ADT, can be bypassed by the local conversion of adrenal androgens to testosterone and de novo local synthesis of androgens through increased expression of enzymes involved in steroidogenesis, such as CYP17A, in the prostatic tissue. The elevated intra-tumor level of androgens stimulates AR paracrine and autocrine activation, regardless of serum androgen levels^[84,85]^.

The intra-tumor synthesis of steroid hormones was shown to be significantly increased in CRPC patients compared to naïve primary PCa. To compare the levels of androgen, a study utilized autopsies and tissue biopsies from hormone-sensitive and hormone-resistant patient tumors, and the latter was shown to possess high levels of continuous androgen production compared to primary tumors^[83]^. A primary source of these de novo intra-prostatic androgens are the adrenal androgens dehydroepiandrosterone (DHEA) and androstenedione (AD), generated by the action CYP17A1 enzyme. In the prostate, DHEA and AD can act on the AR axis once converted to testosterone and DHT. DHEA also exists predominantly in a sulfated form (DHEA-S). In CRPC, although ABI (CYP17 inhibitor) results in a significant decline of both DHEA and AD, the persistent DHEA-S can serve as a precursor for testosterone and DHT synthesis in prostate tissue, thus conferring resistance to ABI^[86-88]^. Studies in CRPC xenografts have shown that several genes involved in the androgen synthesis pathway are upregulated during hormonal therapy. The overexpression of AKR1C3, an enzyme implicated in the steroidogenesis pathway, drives resistance to ABI acetate via increasing androgen synthesis and signaling^[89]^. In line, downregulating AKR1C3 re-sensitizes resistant cells to ABI treatment. Consistently, AKR1C3 activation has been shown to be associated with ENZ resistance^[90]^. Other enzymes implicated in steroidogenesis, i.e., SRD5A1 and HSD3B2, were found to be overexpressed in samples from CRPC bone marrow metastases^[91]^.

### Glucocorticoid receptor overexpression

The role of glucocorticoids is complex in PCa because of their dual role. Despite that, it is shown that whenever adenosine diphosphate (ADP) is used to antagonize AR signaling, the GR is activated and overexpressed to confer resistance to the treatment used^[92]^. Both GR and AR have similar structures and mechanisms of action; thus, in mCRPC, whenever a treatment is used to block AR signaling^[93]^, GR is usually activated to attach to nuclear AREs and activate genes that stimulate tumor progression and cell endurance^[94]^. A preclinical study showed that GR was overexpressed and conferred resistance in *in vitro* cell lines (VCaP and LNCaP/AR) that were treated with apalutamide and ENZ. However, it was shown that VCaP was desensitized to ENZ when GR was knocked out^[93]^. Another study also showed that CRPC was resistant to DOC when GR was overexpressed. In this study, GR antagonism was used to successfully re-sensitize the cells to DOC^[95]^. Puhr *et al*.^[96]^ observed that GR is expressed minimally in primary PC tissue and the expression notably increases with long-term treatment with ENZ.

### Neuroendocrine differentiation

While only about 1% of all primary PCa are diagnosed as neuroendocrine prostate cancer (NEPC), up to 30% of mCRPCs are NEPC^[97]^. This profound phenotypic shift, as a result of selection pressure from ADT or potent antiandrogens, from tumors with histological features of adenocarcinoma that express AR to AR-negative neuroendocrine prostate tumors has been termed lineage plasticity^[98]^. The loss or mutation of both tumor suppressors TP53 and RB1 has been found to be crucial in NEPC differentiation^[99]^. Tan *et al*.^[100]^ observed Rb protein loss in 90% of NEPC with RB1 allelic loss in 85% of cases. A recent *in vivo* preclinical mouse study found that *Rb1* loss promotes plasticity and metastasis in PCa with TP53, and tumor suppressor phosphatase and tensin homolog* (Pten)* loss causes secondary resistance to therapies that target the AR signal axis^[101]^. A different preclinical study of *in vitro* and *in vivo* models of human PCa showed evidence of lineage plasticity and a shift from androgen-dependent PCa to androgen-independent NEPC after treatment with ENZ^[102]^. This phenotypic shift was induced by the loss of RB1 and TP53 and was facilitated through overexpression of the transcription factor SOX2. It was also demonstrated that the inhibition of SOX2 restored the function of TP53 and RB1^[102]^. At the moment, there are no therapeutics in drug development studies that target the loss or mutations of TP53 or RB1. However, a Phase II study is currently ongoing to evaluate the potential use of alisertib (aurora kinase A inhibitor) in mCRPC and NEPC patients^[103]^.

### Tumor microenvironment

The ability of the tumor microenvironment to promote drug resistance is likely linked to the role of the cancer-associated stromal cells in tumor initiation and progression. Tumor microenvironment-derived neuregulin 1 (NRG1), identified in cancer-associated fibroblast (CAF) supernatant, was shown to induce resistance in tumor cells through activation of human epidermal growth factor receptor-3 (HER3) and the subsequent downstream signaling molecules such as MAPK to promote cell proliferation and survival^[104]^. Blocking the NRG1/HER3 axis was shown to re-sensitize tumors to hormone deprivation *in vitro* and *in vivo*. In addition, patients with mCRPC having increased tumor NRG1 activity showed an inferior response to second-generation antiandrogen therapy^[105,106]^. Zhang *et al*.^[106]^ showed that ENZ drives in overproduction of NRG1. It is still unclear how AR inhibitors affect the production of NRG1, but many studies have shown that NRG1 overexpression is correlated with poor outcomes and reaction to ADT.

SPP1, an important extracellular matrix component secreted by multiple kinds of cell types including immune cells, fibroblasts, osteoclasts, smooth muscle, and epithelial cells, is overexpressed in many types of tumors^[107,108]^. In the GSE32269 dataset, SPP1 expression was shown to be significantly higher in the mCRPC group than in the primary PCa group. The Human Cancer Metastasis Database analysis showed that SPP1 expression levels were significantly higher in bone metastases than in lymph node and posterior peritoneum metastases, thus correlating the expression of SPP1 with the progression of mCRPC^[109]^. To investigate the relationship between SPP1 expressions and ENZ resistance *in vivo*, Pang *et al*.^[108]^ studied the effect of ENZ treatment on SPP1 knock downed cells (22Rv1 cell line). The results show that SPP1 knockdown significantly inhibited 22Rv1 cell proliferation after ENZ treatment.

### Other signaling alterations/implication of growth factors, kinases, cytokines, enzymes... 

The aberrant activation of multiple signaling pathways plays a key role in deriving drug resistance in mCRPC. High levels of growth factors including IGF-1, EGF, and TGF-α/β have been reported in mCRPC^[110]^. A study showed that the overexpression and activation of EGFR mediate DOC resistance in CRPC by inducing AKT-dependent ABCB1 (MDR1) expression^[111,112]^. Besides, the stimulation of EGFR was shown to derive the activation of Ack1/Tnk2, which is known to correlate positively with the progression to the mCRPC stage^[113]^. In addition, PCa patients whose tumors showed moderate to strong staining of activated Ack1 displayed a poor prognosis^[113]^.

Several studies assured the association of mCRPC with increased activation of the PI3K-AKT-mTOR pathway^[114,115]^. Phosphatase and Tensin Homolog (PTEN) has been attributed to the radical amelioration of the PI3K/AKT pathway in nearly 50% of PCa patients. This atypical augmentation of the latter pathway distorts the protein-serine/threonine kinases promoting castration-resistant growth. PI3K-AKT-mTOR pathway interacts and cooperates with several key oncogenic signaling cascades such as MAPK and WNT signaling to facilitate PCa growth and drug resistance^[116]^. Furthermore, augmented levels of ERKs activation have been reported in recurrent mCRPC patients, and correlative studies have further linked several mutations down the RAS isoforms with the tumorigeneses of PCa^[117]^. Although collateral activation of the PI3K/AKT and RAS/MAPK pathways have been implicated in several studies, cell proliferation and invasiveness of p63-expressing prostate tumors were solely attributed to the MAPK signaling pathway, which suggests its critical involvement in mCRPC^[118]^.

Cytokines overexpression is also known to trigger tumor progression and drug resistance in CRPC^[119,120]^. A study reported that inhibiting STAT3, a downstream target of IL-6, results in decreased growth of ENZ-resistant cells. Consistently, blocking STAT3 signaling by inhibiting its phosphorylation was shown to re-sensitize ENZ-resistant cells, hence supporting the implication of the IL-6-STAT3 pathway in ENZ-acquired resistance^[121]^. Moreover, radically increased IL-6 expression is a direct consequence of the constitutive activation of the NF-κB pathway^[122,123]^. NF-κB levels influence the expression of an essential PCa biological marker for progression, PSA. Additionally, NF-κB is a key factor in the upregulation of IL-8 cytokine; the latter is involved in the regulation of prostate vasculature and apoptosis^[124]^. High levels of circulating IL-8 were detected in advanced PCa at a stage when the tumors no longer respond to antiandrogens^[125,126]^. PCa cells overexpressing IL-8 show reduced effectiveness of bicalutamide^[124]^. The pro-inflammatory cytokine TNF-α has also been reported to be elevated in CRPC, and such chronic stimulus is a prototypical inducer of NF-κB expression, thereby increasing metastasis, proliferation, and drug resistance^[127]^.

Analysis of whole-exome and transcriptome sequencing of mCRPC biopsies revealed that alterations in DNA-damage repair (DDR) genes including *BRCA2*, *BRCA1*, and *ATM* occur at higher frequencies in mCRPC (19.3%) than in primary PCa, of which 12.7% of the samples were identified with loss of BRCA2^[43]^. Cells with deleterious mutations in *BRCA1* or *BRCA2* compensate for this loss by increasing their dependency on poly(ADP-ribose) polymerase (PARP) activity for DNA repair^[128,129]^.

Prostate-specific membrane antigen (PSMA) is a type II transmembrane glycoprotein that is normally expressed in the prostate epithelium. Although it is expressed in other tissues (such as the salivary glands, proximal tubules of the kidney, and small intestine), its levels there are minimal. Importantly, in PCa tissues, PSMA is significantly overexpressed, having the highest expression in advanced PCa and mCRPC. Moreover, following androgen deprivation and hormonal therapy, PSMA expression seems to be increased^[130-132]^.

## EMERGING TREATMENTS TARGETING mCRPC

Based on the above, there is a crucial need for novel alternative approaches and drugs that could overcome resistance in advanced PCa stages. In fact, several treatments, which are under development and trials, could emerge as promising therapies for patients with mCRPC and become the next-generation standards of care. Table 2 summarizes the evolving targeted treatments in mCRPC along with their relevant clinical trials^[15,133-156]^.

**Table 2:** Summary of the completed and ongoing clinical trials of emerging therapies in mCRPC

**Therapy**	**Agent(s)**	**Trial name/Clinicaltrials.gov identifier**	**Trial phase**	**Main findings/Comments**
**PARPi**	Olaparib	TOPARP-A/NCT01682772^[133]^	II	Olaparib improved the RR specifically in patients with DDR gene defects
	Olaparib	TOPARP-B/NCT01682772^[134]^	II	Olaparib improved the RR specifically in patients with *BRCA1/2 *aberrations
	Olaparib or ENZ/ABI	PROfound/NCT02987543^[15,135]^	III	Olaparib increased PFS and OS
	Rucaparib	TRITON2/NCT02952534^[136,137]^	II	Rucaparib improved RR and PSA RR in patients with* BRCA* alterations
	Rucaparib or physician’s choice of ABI/ENZ/DOC	TRITON3/NCT02975934	III	Ongoing trial
	Niraparib	GALAHAD/NCT02854436^[138,139]^	II	Ongoing trial; interim results show that niraparib improved RR in patients with *BRCA1/2 *biallelic alterations
	Talazoparib	TALAPRO-1/NCT03148795^[140]^	II	Ongoing trial; interim results show that talazoparib improved RR in patients with *BRCA1/2* alterations
	Talazoparib + ENZ or ENZ	TALAPRO-2/NCT03395197	III	Ongoing trial
**PSMA radioligand therapy**	LuPSMA	[141,142]	II	LuPSMA showed high RR, low toxicity, and reduction of pain in patients LuPSMA showed a better response than other therapies when rechallenged upon progression
	LuPSMA or cabazitaxel	TheraP/NCT03392428^[143]^	II	The percentage of patients who achieved PSA_50_ is higher in the LuPSMA group The percentage of patients with AEs is lower in the LuPSMA group
	LuPSMA + best supportive/best standard of care or best supportive/best standard of care	VISION/NCT03511664^[144]^	III	LuPSMA improved PFS and OS
**PSMA-targeted immunotherapy**	CAR^+^ T cells	NCT01140373^[145]^	I	3 10^7^ CAR^+^ T cells/kg was safe with persisting CAR-T cells in peripheral blood for up to two weeks One patient exhibited a long-term response with stable disease status for more than 16 months
	PSMA-targeted/TGFβ-resistant CAR-T cells	NCT03089203^[146]^	I	Ongoing trial
	Fully humanized anti-PSMA monoclonal IgG1 antibody conjugated to MMAE PSMA-ADC	NCT01695044^[147]^	II	Discontinued trial as 40% and 31% of the participants showed progressive disease and AEs, respectively
	MEDI3726 (PSMA-ADC linked to pyrrolobenzodiazepine)	NCT02991911^[148]^	I	AEs occurred in 91% of the patients and 33% discontinued MEDI3726 exhibited responses at higher doses, but treatment was discontinued
	MLN2704 (PSMA-ADC with a humanized monoclonal antibody linked to the maytansinoid DM)	[149]	I/II	MLN2704 had a low PSA_50_ response Neurotoxicity was dose-limiting
	Pasotuxizumab (AMG 212 or BAY 2010112)	NCT01723475^[150]^	I	AMG 212 had dose-dependent clinical efficacy and manageable safety Relatively long-term response was seen in two patients
	Acapatamab (AMG 160)	NCT03792841^[151,152]^	I	Acapatamab had an acceptable safety profile, promising PSA_50_ response, stable disease in 8/15 patients, and 14% of the patients continued the treatment for more than 6 months
**Androgen receptor degraders**	ARV-110	NCT03888612^[153]^	I	Ongoing trial
**PI3K pathway inhibitors**	Ipat (GDC-0068) + ABI or placebo + ABI	NCT01485861^[154,155]^	Ib/II	Ipat improved PFS and OS Patients with PTEN loss had prolonged PFS
	Ipat + ABI or placebo + ABI	NCT03072238^[156]^	III	Ongoing trial; interim results show that Ipat prolonged PFS Two treatment-related deaths occurred in both groups

ABI: Abiraterone; ADC: antibody-drug conjugate; AEs: adverse events; CAR-T cells: chimeric antigen receptor T cells; DDR: DNA-damage repair; DOC: docetaxel; ENZ: enzalutamide; IgG1: immunoglobulin G1; Ipat: ipatasertib; LuPSMA: lutetium-177[^177^Lu]-PSMA-617; MMAE: monomethyl auristatin E; OS: overall survival; PARPi: poly(ADP-ribose) polymerase inhibitor; PFS: progression-free survival; PSA: prostate-specific antigen; PSA_50_: > 50% decline in prostate-specific antigen; PSMA: prostate-specific membrane antigen; RR: response rate; TGFβ: transforming growth factor-β.

### PARP inhibitors

The PARP superfamily, with at least 18 members, comprises nuclear enzymes involved in the DNA repair machinery of single-stranded breaks (SSBs), cell proliferation, and death^[157]^. Inhibition of PARP proteins impedes the ligation of SSBs that consequently convert into double-stranded breaks (DSBs). When DDR genes are defective, PARP inhibition leads to the accumulation of DSBs, promoting an enhanced lethal effect known as the “synthetic lethality” that ultimately induces cell death. According to this rationale, PARP inhibitors (PARPi) emerged as a therapeutic approach targeting malignancies with defective DDR genes, particularly BRCA1/2^[158-160]^. In fact, PARPi application showed success in BRCA-deficient breast and ovarian cancer and promising efficacy in clinical trials involving mCRPC patients^[129,161]^. Several PARPi have been tested and approved in mCRPC including olaparib, rucaparib, niraparib, talazoparib, and veliparib.

Olaparib (Lynparza) is a small, oral, and bioavailable molecule that selectively binds and inhibits PARP^[162]^. Mateo *et al.*^[134]^ published two Phase II clinical trials, “TOPARP-A” (NCT01682772) and “TOPARP-B” (NCT01682772), involving patients with mCRPC who had previously received standard treatments and who were treated with olaparib. Both studies demonstrated that patients with defective DDR genes and who were no longer responding to the current treatments exhibited a high response rate to olaparib. Importantly, response to treatment seemed dependent on specific DDR gene defects, as the highest response was achieved in the BRCA1/2 aberrant subgroup^[133,134,163]^.

In May 2020, based on the results of the “PROfound” (NCT02987543) clinical study, olaparib was granted a “breakthrough” FDA approval for the treatment of men with mCRPC harboring germline and/or somatic mutations in DDR genes and who formerly received second-generation antiandrogens^[164]^. PROfound is a prospective, randomized, Phase III trial. Patients with mCRPC and DDR genes alterations whose disease progressed while receiving ENZ or ABI were randomly assigned to receive either olaparib or ENZ/ABI (control). The results show that patients receiving olaparib had significantly longer median imaging-based PFS than the control group^[135]^. Moreover, this study was the first to show that patients on opalarib had a significantly longer duration of survival compared to the control^[15]^.

Recently, Marshall *et al.*^[165] ^performed a retrospective, observational study involving patients with mCRPC having somatic or germline mutations in *BRCA1*, *BRCA2*, or *ATM* who were treated with olaparib. This study aimed to assess whether responses to olaparib are different between men with mutations in *BRCA1/2 vs*. *ATM*. Indeed, patients with *BRCA1/2 *alterations exclusively achieved > 50% decline in prostate-specific antigen (PSA_50_ response). Moreover, these patients had a longer median PFS. Thus, this study demonstrated that men with *BRCA1/2* respond better to opalarib than those harboring *ATM* mutations, and better alternatives should be considered for patients with ATM alterations^[165]^.

These clinical trials helped to establish the efficacy and safety of opalarib as a monotherapy. Other trials investigating opalarib as a monotherapy outside the scope of DDR genes are underway^[166]^. Moreover, several preclinical and clinical studies testing the synergistic effects of opalarib with other cytotoxic drugs (chemotherapy, AR-directed therapy, immune checkpoint inhibitors, radioligand therapy, radiotherapy, *etc*.) on mCRPC are still ongoing or completed.

Rucaparib is a small, oral, bioavailable PARPi that was evaluated in clinical trials due to its chemosensitization, radiosensitization, and antineoplastic potency^[167]^. Rucaparib elicits a cytotoxic effect comparable to opalarib^[168]^. Results of “TRITON2” (NCT02952534) Phase II trial prompted an “accelerated” FDA approval of rucaparib for men with mCRPC and *BRCA* mutations who were previously treated with AR-directed therapy and taxane-based chemotherapy. In this study, rucaparib treatment on patients with *BRCA* alterations showed promising response rates, particularly in comparison to patients with non-*BRCA* DDR gene alteration^[136,137,169]^.

Based on the *ClinicalTrials.gov *website, a Phase III clinical trial, “TRITON3” (NCT02975934), is ongoing. Patients with mCRPC harboring *BRCA1*, *BRCA2,* or *ATM* mutations who progressed on AR-directed therapy but did not receive chemotherapy are actively being recruited. This study aims to assess the patients’ response to rucaparib monotherapy *vs.* a treatment of the physician’s choice of ABI, ENZ, or DOC to verify the clinical benefit of rucaparib. In addition to TRITON3, other studies are currently active to evaluate the efficacy of rucaparib in combination with nivolumab or chemotherapeutic treatments^[160]^.

Niraparib (Zejula) is a small, oral, once-daily, bioavailable, potent, and highly selective Poly (ADP-ribose) Polymerase Inhibitors(PARPi) with antineoplastic activity^[170]^. “GALAHAD” (NCT02854436) is an active, ongoing, Phase II clinical trial that aims to assess the efficacy, safety, and pharmacokinetics of niraparib in men with treatment-refractory mCRPC and DNA repair alterations (biallelic alterations). Interim analysis shows that niraparib may have promising efficacy as a monotherapy for men with mCRPC, specifically those harboring *BRCA1/2* biallelic alteration^[138,139]^. Niraparib is also being tested in several trials as a combination therapy with other cytotoxic drugs (ABI and radium-223).

Talazoparib (Talzenna) is a small, oral, bioavailable molecule that was demonstrated to be the most potent among PARPi in terms of *in vitro *activity and trapping of PARP on DNA SSBs^[171,172]^. Between October 2017 and March 2020, participants were recruited and enrolled in “TALAPRO-1” (NCT03148795), an ongoing Phase II clinical trial whereby patients with mCRPC and DDR gene alterations who progressed on standard treatments were eligible and provided with talazoparib. In addition, different clinical trials assessing the efficacy of combining talazoparib with other therapeutics are ongoing including the Phase III TALAPRO-2 (NCT03395197) trial, which compares the combination of talazoparib and ENZ *vs*. ENZ alone^[140]^.

### PSMA-based therapies

The overexpression of PSMA, particularly in mCRPC, makes it a potential therapeutic target for PCa. Upon binding to their receptors, PSMA ligands are internalized, leading to persistent retention of the ligand intracellularly. This signaling process seems specific to cancer cells, as in normal cells, a relatively rapid washout takes place. Abusing this feature in cancer cells that have increased PSMA ligand uptake, targeting PSMA by radioligand therapy or by immunotherapy has become an emerging therapeutic approach to treat mCRPC^[173,174]^.

#### PSMA radioligand therapy 

The radioligand targeting PSMA, which has come the farthest in development, is lutetium-177, [^177^Lu]-PSMA-617 (LuPSMA). It is a small, radiolabeled beta-emitter with a high binding affinity to PSMA. LuPSMA is advantageous by the short-range path length of the emitted beta particle, allowing efficient delivery of the radiation to the target cells while minimizing off-target effects on surrounding tissues^[141]^. Since 2014, many retrospective and prospective studies have been performed to assess the efficacy and safety of LuPSMA in treating mCRPC.

The German Society of Nuclear Medicine performed the largest multicenter retrospective data analysis that studied the toxicity and efficacy of LuPSMA on 145 mCRPC patients. This study showed promising results for LuPSMA in terms of high efficacy and desirable safety, whereby 40% of the patients responded after a single treatment cycle^[175]^.

Hofman *et al*.^[141]^ performed a Phase II prospective trial involving men with mCRPC who progressed after receiving conventional PCa treatments and had high expression of PSMA. This trial’s sample size was expanded, and long-term outcomes were assessed by Violet *et al*.^[142]^. Analysis of the results of those studies showed a high response rate, low toxicity, reduction of pain in patients with mCRPC who received LuPSMA, and better response over other therapies when rechallenged upon progression.

The results of two recent trials, the “TheraP” (NCT03392428) Phase II prospective study and the “VISION” (NCT03511664) Phase III trial, involving mCRPC patients treated with LuPSMA proved that the latter is a potential novel efficient therapeutic for mCRPC and possible alternative to the approved chemotherapeutic drug CBZ and taxane-based regimens^[143,144]^.

Finally, although LuPSMA is the most studied PSMA radioligand therapeutic agent in recent years, other molecules are also being investigated, such as the alpha-emitting actinium-225 (225Ac-PSMA-617). This is an alpha-emitter with higher potency and a shorter range than beta-emitters^[176]^. A meta-analysis was recently published in September 2021 that summarizes the effects of 225Ac-PSMA-617 in mCRPC from nine studies with 263 patients; the authors concluded that 225Ac-PSMA-617 might be a potentially effective therapeutic option for mCRPC patients^[177]^.

#### PSMA-targeted immunotherapy

In addition to its significantly high expression in mCRPC, PSMA has a large extracellular domain which makes it an ideal target for immune agents^[174]^. Several approaches for PSMA-targeted immunotherapy exist including chimeric antigen receptor T cells, antibody-drug conjugates, bispecific T-cell engagers, and PSMA-directed vaccines.

a. Chimeric antigen receptor T cells

Chimeric antigen receptor (CAR) is a genetically engineered transgenic T-cell receptor. It consists of an antigen recognition moiety allowing T cells to identify intact specific tumor-associated antigens and T-cell signaling domains that activate T cells. Thus, CAR-T cells combine the properties of antibodies that recognize particular antigens with the cytolytic killing of T cells^[178-180]^.

A Phase I study (NCT01140373) was performed by Slovin *et al*.^[145] ^to assess the safety, tolerability, and efficiency of escalating doses of PSMA-targeted CAR-T cells. Seven patients included in this study received from 10^7^ to 3 10^7^ CAR-T cells/kg. The highest given dose was shown to be safe with persisting CAR-T cells in peripheral blood for up to two weeks. Importantly, one patient exhibited a long-term response having a stable disease status for more than 16 months^[145]^.

Moreover, a first-time Phase I clinical trial (NCT03089203) to test the safety, feasibility, and efficacy of PSMA-targeted/TGFβ-resistant CAR-T cells is ongoing. The experimental approach strives to overcome the immunosuppressive tumor microenvironment (TME) in mCRPC, to which high levels of TGFβ are a contributing factor. This rationale is based on *in vivo* disseminated PCa models results, where PSMA-redirected CAR-T cells expressing a dominant-negative TGFβ receptor had an increased T-cell proliferation and cytokine release, long-term persistence, and greater tumor elimination^[146]^. The results of this clinical trial are pending.

b. Antibody-drug conjugate

Antibody-drug conjugate (ADC) technology is an emerging therapeutic approach that is still in its early phases. ADCs are highly specific monoclonal antibodies targeted against specific tumor antigens and chemically conjugated to cytotoxic agents^[181]^. Target selection is one of the key considerations in ADCs design. The target needs to be highly expressed on the surface of tumor cells *vs. *low to no expression in normal cells. Moreover, the target preferably needs to have internalization characteristics that allow the transport of the antibody to carry the cytotoxic payloads into cancerous cells. These requirements are met in PSMA, making it an attractive target; therefore, several ADCs targeting PSMA in PCa are in clinical development^[182]^.

A Phase II trial (NCT01695044) was performed by Petrylak *et al.*^[147]^, a Phase I trial (NCT02991911) by de Bono *et al*.^[148]^, and a Phase I/II multiple escalating dose trial by Milowsky *et al*.^[149]^ tested different PSMA-ADCs. The different trials showed that, although these drug conjugates had some activity in mCRPC, their effect was accompanied by significant adverse events (such as neutropenia and neuropathy), which led to the discontinuation of some of these treatments. Hence, it was noticed that toxicity related to the long circulation in the system of PSMA-targeted ADCs limited the success of the clinical trials^[183]^.

c. Bispecific T-cell engagers

Bispecific T-cell engagers (BiTEs) are bispecific antibodies that simultaneously target a T-cell-specific molecule (almost always CD3 chain due to its conserved property) and a TAA, which could be PSMA in the case of PCa^[184]^. BiTEs showed success in several types of cancer, such as acute lymphoblastic leukemia, where blinatumomab was the first FDA-approved BiTE^[185,186]^.

Hummel *et al*.^[150] ^conducted a first-in-human, dose-escalation, Phase I clinical trial (NCT01723475) to primarily test PSMA CD3 first-generation BiTE pasotuxizumab (known as AMG 212 or BAY 2010112) in patients with mCRPC refractory to standard therapy. In this study, data analysis demonstrated the dose-dependent clinical efficacy and manageable safety of pasotuxizumab in mCRPC, with 3 out of 16 patients exhibiting ≥ 50% PSA response and two patients having a relatively long-term response^[150]^.

Another first-in-human, Phase I study (NCT03792841) to assess acapatamab (AMG 160), a novel next-generation PSMA CD3 BiTE with an extended half-life, in mCRPC was conducted^[151]^. AMG 160 gave a promising PSA_50_ response and acceptable safety profile in treated patients. This study prompted testing AMG 160 as a combination therapy with other agents (pembrolizumab, ENZ, ABI, AMG 404, and etanercept prophylaxis) in ongoing trials^[152,187,188]^.

d. PSMA-directed vaccines

Cancer vaccines, intended to enhance the immune response against tumor cells by increasing the pool of TAA-specific host T cells, have failed to demonstrate a considerable benefit in PCa thus far (such as the Phase III clinical trials “PROSTVAC” that targets PSA and “GVAX”, where both failed to meet their primary endpoint of enhancing the overall survival)^[189,190]^. However, this approach continues to be investigated, given its potential efficacy, minimal side effects, limited cost, and easy synthesis. Specifically, PSMA is being considered as an appropriate target for the development of PCa vaccines^[191]^. Using new computational tools to select suitable B-cell epitopes with high antigenicity, the “673RHVIYAPSSHNKYAGE25” peptide was predicted as having the best binding affinity. Future steps involve synthesis of the peptide and testing its *in vivo* efficacy as a potential PSMA-directed vaccine^[192]^.

### Androgen receptor degraders

Androgen receptor degraders emerged as an alternative therapeutic strategy to manage mCRPC. One of the tools that allow targeting of AR to degradation is using proteolysis-targeting chimera (PROTAC) novel technology. PROTACs, also known as bivalent chemical protein degraders, are heterobifunctional compounds consisting of two recruiting ligands joined by a linker. One ligand moiety binds to the protein of interest, while the other is specific to E3 ubiquitin ligase (E3). Thus, PROTACs form a ternary complex with the target protein and E3 and consequently promote target ubiquitination and degradation^[193]^.

Salami *et al*.^[194]^ published a study in 2018 that aimed to assess whether ARCC-4, a low-nanomolar PROTAC ARD, was better at targeting AR signaling in CRPC cells than the currently approved competitive antagonist ENZ. Interestingly, ARCC-4 promoted the degradation of about 95% of cellular ARs, especially the clinically relevant AR with point mutations resistant to ENZ. Moreover, ARCC-4 induced apoptosis and maintained its antiproliferative effect in a hyperandrogenic environment that impedes ENZ activity. This study showed promising results where PROTAC-mediated AR degradation might overcome the mCRPC AR-dependent mechanism of drug resistance^[194]^.

To date, several PROTACs reached clinical trials in different types of cancer, including ARV-110 in mCRPC. The oral and bioavailable ARV-110 was tested earlier *in vitro *and* in vivo*, where it led to the degradation of more than 95% of ARs in the tested PCa cell lines and demonstrated antitumor effects in both ENZ-naïve and -resistant PCa xenograft models. ARV-110, similar to ARCC-4, promoted the degradation of clinically relevant AR mutants and maintained its activity even in a milieu with high androgen levels. The first-in-human, Phase I clinical trial (NCT03888612) testing ARV-110 in mCRPC patients who received more than two prior treatments was performed. This trial is still ongoing; however, early data show that ARV-110 has a reasonable safety profile and a promising antitumor activity in mCRPC^[153]^.

The results from these preclinical and clinical trials, among others, show that ARD might be a novel alternative therapeutic option to treat mCRPC.

### PI3K pathway inhibitors

As mentioned above, loss of PTEN and the atypical activation of the PI3K signaling network are one of the possible mechanisms driving mCRPC growth. This fact supported the development of several PI3K pathway inhibitors including ipatasertib (Ipat) (GDC-0068), which is an oral, bioavailable, AKT non-ATP-competitive inhibitor that impedes the PI3K/AKT pathway and subsequently tumorigenesis^[195]^.

Two clinical trials, a Phase Ib/II (NCT01485861) involving patients who formerly received DOC and an ongoing Phase III trial (NCT03072238) with treatment-naïve patients, tested the combination of Ipat and ABI in mCRPC. In general, both studies demonstrated enhanced antitumor effects when combining Ipat with ABI compared to ABI monotherapy, with increased benefit for patients with PTEN loss^[154-156]^. Thus, the results of those trials suggest that a combination of AKT and AR signaling inhibitors might provide a promising therapeutic approach to treating men with mCRPC with a poor prognosis due to PTEN loss.

## CONCLUSION

mCRPC remains a challenge for PCa disease management. AR signaling comprises a fundamental role in PCa pathogenesis even in the advanced androgen-insensitive stages. AR signaling, along with other various molecular pathways, is subjected to diverse modifications and aberrations that can be used as biomarkers to personalize treatment for mCRPC patients and overcome drug resistance to the standard therapy. Thus, further understanding of the mechanisms mediating drug resistance in mCRPC is crucial for identifying future targeted therapeutic modalities.

## DECLARATIONS

### Copyright

© The Author(s) 2022.
